# Effects of Modified Simiao Decoction on IL-1**β** and TNF**α** Secretion in Monocytic THP-1 Cells with Monosodium Urate Crystals-Induced Inflammation

**DOI:** 10.1155/2014/406816

**Published:** 2014-06-05

**Authors:** Ya-Fei Liu, Sheng-Hao Tu, Zhe Chen, Yu Wang, Yong-Hong Hu, Hui Dong

**Affiliations:** Institute of Integrated Traditional Chinese and Western Medicine, Tongji Hospital, Tongji Medical College, Huazhong University of Science and Technology, 1095 Jiefang Avenue, Wuhan, Hubei 430030, China

## Abstract

Simiao pill, a Chinese herbal formula containing four herbs, has been used in the treatment of gouty arthritis for many years. The aim of this study was to explore the effects of modified Simiao decoction (MSD) on IL-1**β** and TNF**α** secretion in monocytic THP-1 cells with monosodium urate (MSU) crystals-induced inflammation. The MSU crystals-induced inflammation model in THP-1 cells was successfully established by the stimulation of phorbol 12-myristate 13-acetate (PMA) and MSU crystals. Then, the MSD-derived serum or control serum extracted from rat was administered to different treatment groups. The morphology of MSU crystals and THP-1 cells was observed. IL-1**β** and TNF**α** protein expression in supernatant of THP-1 cells were determined by ELISA. Our data demonstrated that MSU crystals induced time-dependent increase of IL-1**β** and TNF**α**. Moreover, MSD significantly decreased IL-1**β** release in THP-1 cells with MSU crystals-induced inflammation. These results suggest that MSD is promising in the treatment of MSU crystals-induced inflammation in THP-1 cells. MSD may act as an anti-IL-1 agent in treating gout. The underlying mechanism may be related to NALP3 inflammasome which needs to be validated in future studies.

## 1. Introduction

Gout, one of the most common inflammatory arthritis in men, is characterized by hyperuricaemia and deposition of monosodium urate (MSU). The annual incidence of gout was ⁡1.6‰ in men and ⁡0.3‰ in women in people over 50 in the United States [1]. With the changes in lifestyle and the rise of obesity, the incidence and complication are increasing.

Despite advances in the application of antigout drugs for the treatment of gout, allopurinol could cause severe hypersensitivity and was restricted in patients with renal insufficiency [2–4]. Colchicine's toxicity was wildly reported [5] and Food and Drug Administration exercised enforcement action against companies illegally marketing unapproved single-ingredient oral colchicine [6]. Therefore, it is urgent to explore new available antigout agents, especially herbal medicine. Plant-based medicines are widely applied to treat gout and its complications in a number of hospitals in China. In Chinese medicine, gout is associated with dampness, heat, sputum, and stasis. Among effective prescriptions, Simiao pill, which was derived from Ermiao powder and described in a famous traditional Chinese medicine monograph Chengfang Biandu in Qing Dynasty of China, has been wildly applied for treatment of gout and inflammatory arthritis. It is composed of four individual herbs:* rhizoma atractylodis*,* cortex phellodendri*,* radix achyranthis bidentatae,* and* semen coicis*. Simiao pill and its associated prescriptions showed their promising efficacy in treating hyperuricemia and gouty arthritis [7–11]. To cope with the complicated pathologic states of gout in different stages, a modified Simiao decoction (MSD) has been developed based on clinical research, clinical experience, and traditional Chinese medicine theory.

MSU was identified as the aetiological agent of gout in the 18th century [12] and shown to be the causative agent of gout in 1848 [13]. Proinflammatory cytokines, such as interleukin-(IL-) 1*β* and tumour necrosis factor (TNF) *α*, play a critical role in orchestrating the acute gouty inflammation triggered by MSU crystals [14]. Recent reports found that MSU involved the activation of NALP3 inflammasome, resulting in the production of IL-1*β* [15–17]. IL-1*β* was produced in monocytes by MSU crystals in vitro [18, 19], and it was recently identified as a key cytokine in gout. Meanwhile, a rapid response was observed in patients with acute gouty arthritis after treatment with anti-IL-1 agents (rilonacept and anakinra) [20, 21]. The study is to explore therapeutic effects of MSD on IL-1*β* and TNF*α* in THP-1 cells with MSU crystals-induced inflammation and to provide evidence for its use in gouty arthritis.

## 2. Materials and Methods

### 2.1. Reagents and Main Devices

Human IL-1*β* (Cat: EHC002b) and TNF*α* (Cat: EHC103a) enzyme-linked immunosorbent assay (ELISA) kits were purchased from Beijing NeoBioscience Technology Co., Ltd., China. Hyclone RPMI 1640 medium (SH30809.01B) was from Thermo Fisher scientific Co., Ltd. (Beijing, China). FBS was purchased from Hangzhou Sijiqing Biological Engineering Materials Co., Ltd. (Hangzhou, China). Uric acid sodium salt (U2875) and phorbol 12-myristate 13-acetate (PMA) (product number: 79346) were ordered from SIGMA-ALDRICH, Co. (St. Louis, USA); Nikon Microimaging System (TE2000-U, Tokyo, Japan); Microplate reader (BioTek Synergy2, Vermont, USA); Inverted microscope (CKX-31, Olympus Corporation, Tokyo, Japan); CO_2_ incubator (New Brunswick Scientific Co., Inc., New Jersey, USA); Esco Airstream Class II Biological Safety Cabinet (Beijing, China); Transmission electron microscope (FEI Tecnai G^2^12, Holland); Rotavapor (BUCHI, Flawil, Switzerland).

### 2.2. Preparation of MSD

Herbal formula MSD is composed of eleven crude herbs which are prepared as seen in Table 1. The rule of compositions is based on traditional Chinese medicinal theory, and the compatibility of herbs is due to our clinical experience. All herbs were purchased from Tongji Hospital in Hubei Province (Wuhan, China) and identified by the Department of Pharmacognosy, Hubei University of Chinese Medicine (Wuhan, China). The rat doses of MSD were converted from human doses (Chinese Pharmacopeia, 2010) based on body surface areas. All herbs were soaked 30 minutes before boiled. The decoction was concentrated by Rotavapor.

### 2.3. Animals and Grouping

Seven-week-old weight 220∼250 g male Wistar rats (*n* = 24), SPF grade were provided by the Center for Disease Control and Prevention of Hubei province (the animal certificate SCXK number 2008-0005) and fed in the barrier system according to The Guidelines for the Care and Use of Animals in Research enforced by Hubei Municipal Science and Technology Commission. All protocols were approved by the Institutional Animal Care and Ethics Committee of Tongji Medical College, Huazhong University of Science and Technology. Food and water were given ad libitum throughout the experiment. The rats were caged in a standard barrier system with a 12-h light/dark cycle. After 7 days of acclimation, the animals were randomly divided into two groups (*n* = 12): one control group and one MSD treatment group (33.6 g/(kg day)).

### 2.4. Administration of MSD

The treatment groups of rats (*n* = 12) were administered with MSD, intragastrically for total seven doses. Oral gavage was performed twice a day. Rats in normal control group were orally administered with the same volume of distilled water. The rats were fasted for 12 h but permitted water ad libitum before blood collection.

### 2.5. The MSD-Derived Serum Preparation

At sixty minutes after the last intragastric administration of MSD, the rats were narcotized with 10% chloral hydrate by intraperitoneal injection. Blood was extracted from aorta abdominalis. After being placed at room temperature for an hour, blood was centrifuged at 3000 ×g for 20 min. Both MSD-containing serum and control serum were filtered by 0.22 *μ*m filter membrane, termed as MSD-S and CONT-S, respectively, and followed by storage at −80°C until application.

### 2.6. Cell Culture

Monocytic THP-1 cells, human monocyte line, obtained as a gift from the Department of Immunology (Tongji Medical College, Huazhong University of Science and Technology), were grown in RPMI-1640 medium supplemented with 10% heat-inactivated fetal bovine serum at 37°C and 5% CO_2_. THP-1 cells were plated at the density of 1.0–1.5 × 10^6^/mL in 6-well plates. THP-1 cells were stimulated for 3 h with 100 ng/mL PMA the day before stimulation. This treatment enhances the phagocytic properties of the cells and prompts a constitutive production of pro-IL-1*β* [17]. THP-1 cells were stimulated with 100 *μ*g/mL MSU in the presence or absence of MSD-S. The prepared MSU solution should be kept at 4°C about one week before forming MSU crystals. THP-1 cells were randomized into normal group (N), model group (M), and treatment group.

### 2.7. ELISA for IL-1*β* and TNF*α* Protein Expression in Supernatant

The production of IL-1*β* and TNF*α* was detected by quantitative sandwich enzyme immunoassay technique according to the manufacturers' standard protocols. The sensitivity of the ELISA kits of IL-1*β* and TNF*α* was 4 pg/mL and 8 pg/mL. None of the samples examined had a cytokine level > 1000 pg/mL. The interassay and intra-assay coefficients of variation of the ELISA kits for IL-1*β* and TNF*α* were less than 10% and 9.5%.

### 2.8. Statistical Analysis

All data with a normal distribution were presented as mean ± standard deviation (SD) and analysed with aid of SPSS17.0 Statistical Software. Statistical significance was determined by one-way analysis of variance (ANOVA). For data with equal variances assumed, ANOVA followed by LSD test was applied. For data with equal variances not assumed, ANOVA followed by Dunnett's T3 test was used. A probability of less than 0.05 was considered to be statistically significant.

## 3. Results

### 3.1. Morphological Characteristics of MSU Crystals

**Figure 1:**
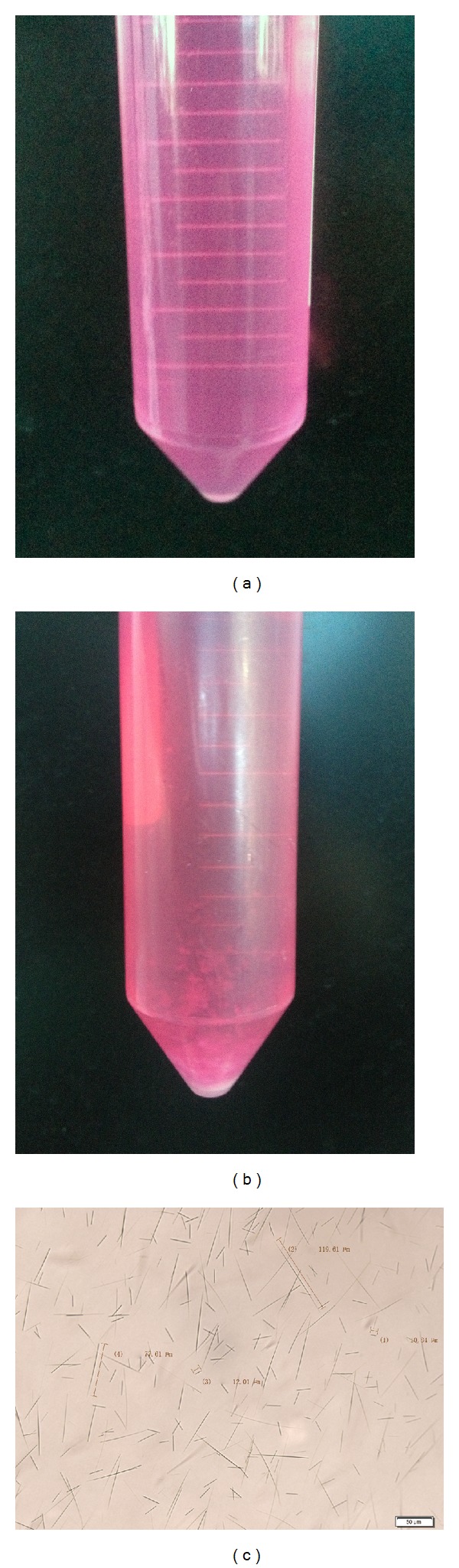
Morphological characteristics of MSU crystals. Immediately prepared 100 *μ*g/mL MSU solution which was dissolved in RPMI-1640 medium (a), the above solution was kept at 4°C about one week ((b), (c)).

Compared with Figure 1(a), cloud-shaped precipitation could be seen in Figure 1(b) (100 *μ*g/mL MSU solution which was dissolved in RPMI-1640 medium). The solution of Figure 1(b) indicated needle-shaped crystals under inverted microscope in Figure 1(c). Crystals produced were 10.84–119.61 *μ*m long (Figure 1(c)).

### 3.2. Morphological Characteristics of THP-1 Cells

**Figure 2:**
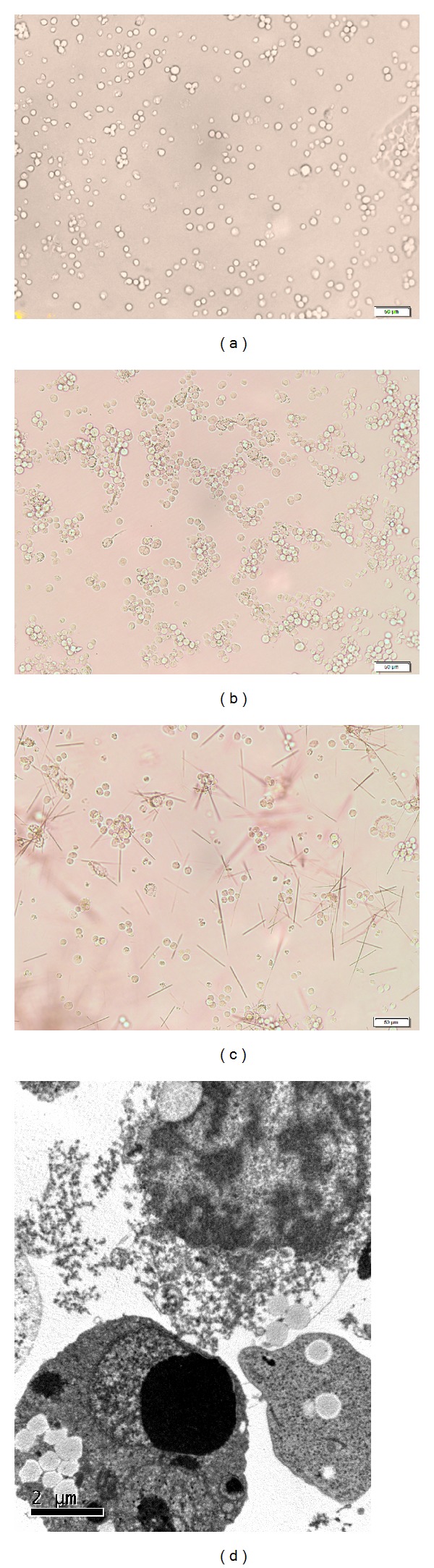
Morphological characteristics of THP-1 cells. Normal THP-1 cells (a); THP-1 cells were stimulated by 100 ng/mL PMA for 3 h (b). After stimulation with PMA, THP-1 cells were stimulated by 100 *μ*g/mL MSU ((c), (d)).

Compared with Figure 2(a), most of THP-1 cells were transformed into cells with the characteristics of mature macrophages after stimulation with 100 ng/mL PMA in Figure 2(b) [22]. After stimulation with MSU, we found many cell lysates under inverted microscope and transmission electron microscope in Figures 2(c) and 2(d). However, THP-1 cells did not show evidence of phagocytosis of MSU crystals after 24 h of culture as determined by transmission electron microscope.

### 3.3. The Effects of MSU on the Production of IL-1*β* and TNF*α*

**Figure 3:**
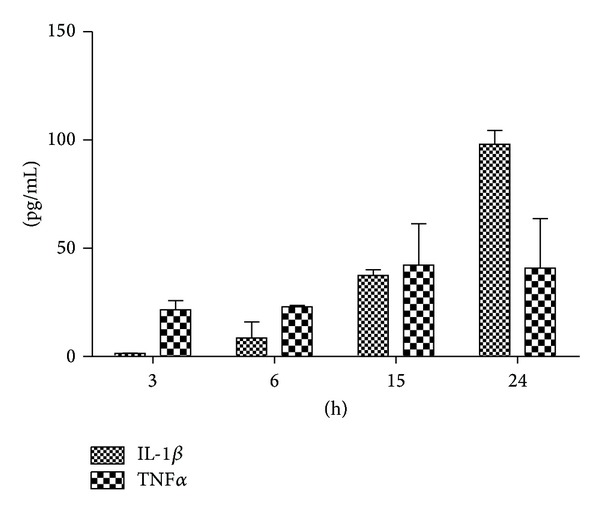
The effects of MSU on the production of IL-1*β* and TNF*α*. Values are mean ± standard deviation (SD). THP-1 cells were stimulated with MSU for the indicated times. Supernatants were analysed for IL-1*β* and TNF*α* production by ELISA.

As shown in Figure 3, IL-1*β* level in cell culture supernatants was time-dependent increase in four time points. TNF*α* level in cell culture supernatants reached peak at 15 h. There was correlation between time point and IL-1*β* level (Pearson correlation coefficient = 0.932, *P* = 0.001) while there was no correlation for TNF*α*. The expression of IL-1*β* in supernatants was distinctly higher than TNF*α* at 24 h.

### 3.4. The Effects of MSD on the Production of IL-1*β*

**Figure 4:**
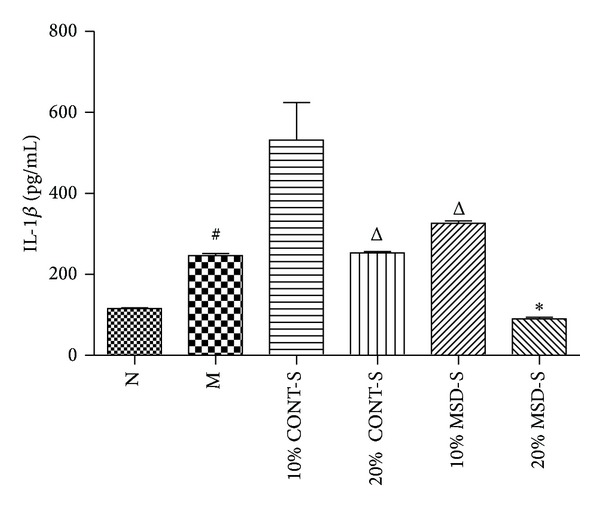
The effects of MSD on the production of IL-1*β* in supernatants of THP-1 cells. Values are mean ± SD. ^#^ *P* < 0.05 compared with group N, *P < 0.05 compared with group M, and ^Δ^ *P* < 0.05 compared with 20% MSD-S. MSD: modified Simiao decoction; MSD-S: MSD-containing serum; CONT-S: control serum; N: normal group; M: model group; 10% CONT-S, 20% CONT-S, 10% MSD-S and 20% MSD-S: treatment group.

As shown in Figure 4, IL-1*β* level in cell culture supernatants was significantly higher in group M than that in N (*P* < 0.05). Compared with group M, there was a significant reduction in the expression of IL-1*β* in group 20% MSD-S. And there was a significant reduction in the expression of IL-1*β* in group 20% MSD-S when compared with groups 10% MSD-S and 20% CONT-S (*P* < 0.05). However, there was no significant reduction in the expression of IL-1*β* in groups 10% MSD-S, 10% CONT-S, and 20% CONT-S when compared with group M.

### 3.5. The Effects of MSD on the Production of TNF*α*

**Figure 5:**
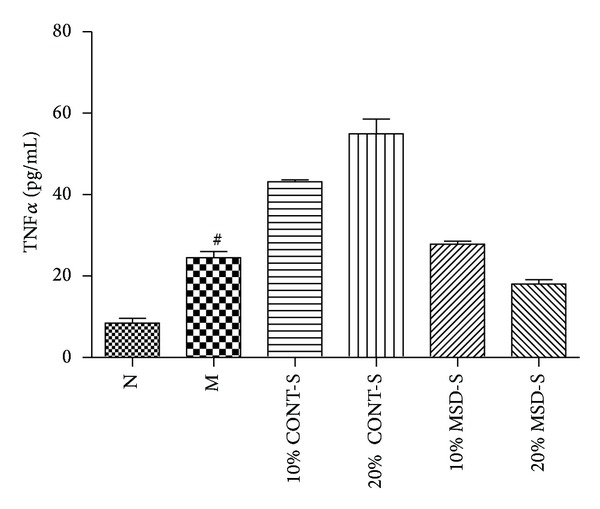
The effects of MSD on the production of TNF*α* in supernatants of THP-1 cells. Values are mean ± SD. ^#^ *P* < 0.05 compared with group N. MSD: modified Simiao decoction; MSD-S: MSD-containing serum; CONT-S: control serum; N: normal group; M: model group; 10% CONT-S, 20% CONT-S, 10% MSD-S and 20% MSD-S: treatment group.

As shown in Figure 5, TNF*α* level in cell culture supernatants was significantly higher in group M than that in N (*P* < 0.05). However, there was no significant reduction in the expression of TNF*α* in all treatment groups when compared with group M.

## 4. Discussion

In traditional pharmacologic studies of traditional Chinese medicine, crude drugs or crude drug compounds are directly added into the culture system of cells or organs in vitro. However, the compositions of traditional Chinese medicine or drug compound are complex and many of the compositions are ineffective until they undergo a series of biotransformations after digestion and absorption in the gastrointestinal tract. Additionally, the pervasion pressure, the pH, the physical and chemical characteristics, and the impurities of traditional Chinese medicine are likely to cause the changes of physiology of reaction system in vitro, thus influencing the validity of experimental results [23]. “Serum pharmacology” was first put forward by Tashino in 1984 [24]. Traditional Chinese medicine is orally administered to animals, blood is collected to separate the serum, and then drug serum is applied for experimental analysis in vitro. Serum pharmacology has been wildly used for pharmacology studies of traditional Chinese medicine in vitro [25–27].

Chinese medicine is identified as an excellent alternative and complementary medicine in treating gout [28]. In traditional Chinese medicine, gout falls into the category of Bi syndrome caused by wind, cold, and dampness which was described in the Yellow Emperor's Classic of Internal Medicine. Previous evidence from clinical practice and experimental studies has confirmed that MSD has the potential to treat hyperuricemia and gouty arthritis [7, 8, 10, 29]. However, previous studies focused on urate lowing therapy. To our knowledge, gouty arthritis could attack in patients with a serum uric acid concentration in the normal range. Ankle joint urate arthritis provided a useful tool for the evaluation of anti-inflammatory and antigout agents [30, 31]. MSU crystals-induced inflammation in monocytes was rarely reported [17]. Our results were consistent with Martinon et al. in terms of time-dependent increase in IL-1*β* and TNF*α* release [17]. The IL-1*β* and TNF*α* release in our study was markedly lower than that in Martinon et al. [17]. The difference may result from sorts of different experimental condition.

The prepared 100 *μ*g/mL MSU solution is supposed to be stored at 4°C about one week before forming cloud-shaped precipitation. Otherwise, MSU crystal cannot be formed. The crystallization of monosodium urate is a complex process which includes growth and nucleation which is a process of new microcrystal precipitation [32–35]. Without monosodium urate crystallization, the production of IL-1*β* and TNF*α* was very low. The production of IL-1*β* and TNF*α* may be due to MSU crystal phagocytosis by monocytes or macrophages [19, 36]. In our study, THP-1 cells did not show evidence of phagocytosis of MSU crystals using transmission electron microscope. The specimen processing prior to taking photos may remove MSU crystals, which leads to our negative result.

In our study, the MSU crystals-induced inflammation model in THP-1 cells was successfully established by the stimulation of PMA and MSU. We demonstrated that 20% MSD-S significantly reduce the expression of IL-1*β*. This may account for the effect of MSD in treating gout. However, other groups showed no effect in lowering IL-1*β* secretion. In animal experiments, MSD also showed the effect of diminishing IL-1*β* secretion [37, 38]. In addition, our study indicated that MSD-S should be maintained at higher concentration to function as an anti-inflammatory agent.

We did not supply evidence of all treatment groups in decreasing the production of TNF*α*. However, two animal experiments showed opposite results with ours [38, 39]. The difference may originate from different models and reagents.

In summary, our study demonstrates that MSU crystals induce time-dependent increase of IL-1*β* and TNF*α*. Moreover, MSD significantly reduces IL-1*β* release in THP-1 cells with MSU crystals-induced inflammation. These results show that MSD is promising in treating MSU crystals-induced inflammation in THP-1 cells. MSD may function as an herbal anti-IL-1 agent in treating gout. The mechanism may be related to NALP3 inflammasome and should be validated in future studies.

## Figures and Tables

**Table 1:** The composition of herbal formula MSD.

Crude herbs	Content	Main components
Rhizome of Chinese atractylode (*rhizoma atractylodis*)	12	Hinesol; atractylone
Amur corktree bark (*cortex phellodendri*)	12	Berberine; palmatine
Twotooth achyranthes root (*radix achyranthis bidentatae*)	12	Inokosterone
Coix seed (*semen coicis*)	30	Coixol; coixenolide
Glabrous greenbrier rhizome (*rhizoma smilacis glabrae*)	30	Dihydroflavonol; astilbin
Seven yam rhizoma (*rhizome dioscoreae septemlobae*)	12	Diosgenin
Plantain seed (*herba plantaginis*)	12	Plantaginin
White mustard seed (*Semen brassicae*)	12	Sinalbin; sinapine
Chinese rhubarb (*radix et rhizome rhei*)	6	Emodin
Largehead atractylodes rhizome (*rhizoma atractylodis macrocephalae*)	15	Atractylon
Hawthorn fruit (*fructus crataegi*)	12	Rutin; vitexin

MSD: modified Simiao decoction.
